# A case of left leg necrotizing fasciitis caused by *streptococcus pyogenes* in a healthy Japanese man

**DOI:** 10.1016/j.idcr.2023.e01775

**Published:** 2023-04-26

**Authors:** Miyu Takagi, Takaaki Kobayashi, Akina Fukushima, Sandra Moody, Akihito Yoshida

**Affiliations:** a Department of General Internal Medicine, Kameda Medical Center, Kamogawa, Chiba, Japan; b Division of Infectious Diseases, University of Iowa, Iowa City, IA, USA; c Department of Medicine, Divisions of Hospital Medicine & Geriatrics, University of California, San Francisco, USA

**Keywords:** Necrotizing fasciitis, *streptococcus pyogenes*

## Abstract

• We experienced a case of necrotizing fasciitis (NF) due to Group A streptococcus in a healthy Japanese man.
• Cutaneous manifestations with NF can be initially minimal.
• It is important to recognize that one of the characteristic symptoms of NF is severe pain out of proportion.
• When NF is suspect, emergent surgical exploration and debridement are required.

## Case illustrated

**Fig. 1:**
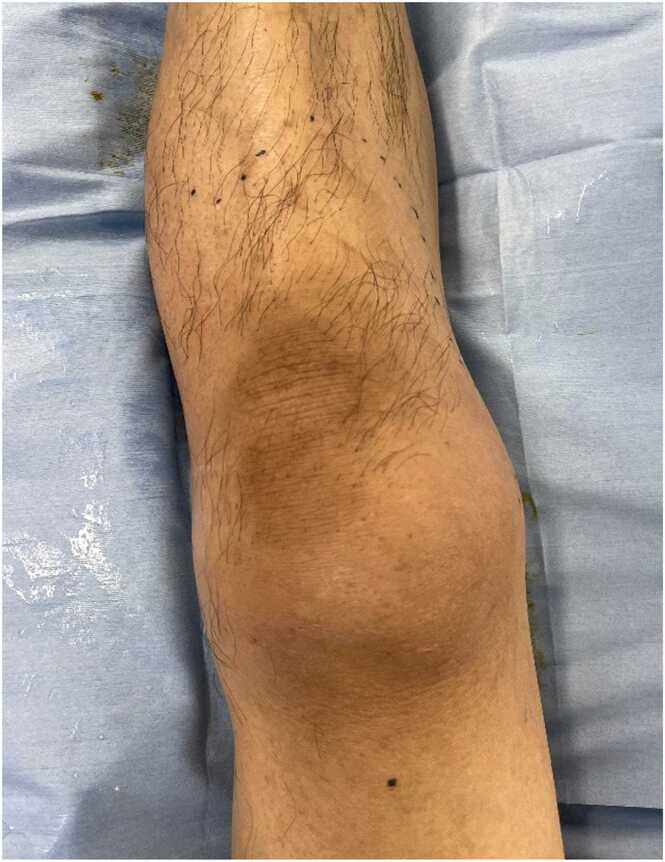
There was mild redness from the left thigh to lower leg with no joint swelling. While redness was mainly over his knee, he had severe tenderness up to the middle of his left shin.

**Fig. 2:**
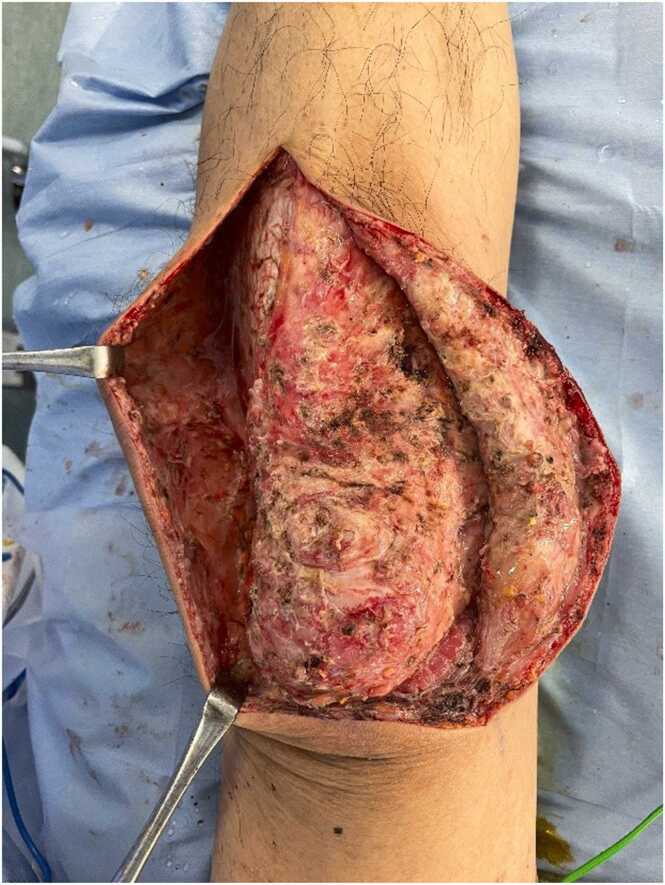
Emergent surgical exploration revealed significant tissue necrosis and small vessel thrombi.

A 50-year-old man with no past medical history presented with left leg pain. One day prior to admission, he noticed a gradual onset of left leg pain while working at a construction site. On the day of admission, the pain became progressively worse and was associated with anorexia and rigors. He denied sore throat or recent trauma. On physical examination, his temperature was 38.4 °C, blood pressure 115/67 mmHg, and heart rate 109 beats per minute. Mild erythema of his left thigh to lower leg was noted (Fig. 1). While tenderness of the left proximal thigh matched the area of erythema, tenderness of the left distal leg exceeded the area of erythema. Computed tomography of the left leg showed swelling in the subcutaneous fatty tissue without obvious gas or abscess. During initial evaluation, his systolic pressure decreased to the 80′s (mmHg). Necrotizing fasciitis (NF) was suspected, and surgical exploration conducted. Necrotic lesions were seen in the deep soft tissue including the fascia, and the peripheral blood vessels were compressed by necrotic lesions (Fig. 2). Emergent debridement was performed, and *streptococcus pyogenes* (GAS) was identified from the culture of the operative specimens. He was successfully treated with penicillin G and clindamycin.

NF is a life-threatening infection that spreads along the fascial planes [1], [2]. Risk factors include diabetes mellitus, liver cirrhosis, obesity, malignancy, heart failure, and kidney disease. However, NF due to GAS may occur among patients without penetrating trauma who have no other risk factor [3]. Since cutaneous manifestations with NF due to GAS can be initially minimal, the diagnosis may be delayed. One of the characteristic symptoms of NF is severe pain out of proportion to exam findings. Early recognition is critical given that a patient with NF is at high risk for systemic toxicity, limb loss and/or death. Treatment for NF consists of surgical debridement and antibiotics therapy. For NF due to GAS, a combination of penicillin and a bacteriostatic, such as clindamycin or linezolid that inhibits toxin production, should be continued for at least 2–3 days [1], [2]. Mortality has been reported to be close to 100% in the absence of surgical debridement [4]. NF should be suspected when a patient complains of pain out of proportion to skin findings, even without trauma or risk factors. When NF is suspected, emergent surgical exploration and debridement are required, given that antibiotics without surgery is associated with a high mortality rate.

## Ethical approval

The local ethical committee approval does not apply in this case.

## Patient consent

The patient’s written consent was obtained.

## Authors’ contributions

MT wrote a first draft of the manuscript. TK critically reviewed and revised the manuscript. All authors read and approved the final paper.

## Funding

None.
